# Deteriorated Dietary Patterns with Regards to Health and Environmental Sustainability among Hungarian Roma Are Not Differentiated from Those of the General Population

**DOI:** 10.3390/nu13030721

**Published:** 2021-02-24

**Authors:** Erand Llanaj, Ferenc Vincze, Zsigmond Kósa, Helga Bárdos, Judit Diószegi, János Sándor, Róza Ádány

**Affiliations:** 1 Department of Public Health and Epidemiology, Faculty of Medicine, University of Debrecen, Kassai street 26/B, H-4028 Debrecen, Hungary; erand.llanaj@med.unideb.hu (E.L.); vincze.ferenc@med.unideb.hu (F.V.); bardos.helga@med.unideb.hu (H.B.); dioszegi.judit@med.unideb.hu (J.D.); sandor.janos@med.unideb.hu (J.S.); 2 Doctoral School of Health Sciences, University of Debrecen, Kassai street 26/B, H-4028 Debrecen, Hungary; 3 Department of Methodology for Health Visitors and Public Health, Faculty of Health, University of Debrecen, Sóstói street 2-4, H-4400 Nyíregyháza, Hungary; kosa.zsigmond@foh.unideb.hu; 4 MTA-DE Public Health Research Group, University of Debrecen, Kassai street 26/B, H-4028 Debrecen, Hungary

**Keywords:** nutrition, Roma, Hungary, health, dietary patterns, dietary indicators, sustainability, dietary recall

## Abstract

Nutritional epidemiology studies on Roma people are scarce and, to date, their nutrient-based dietary patterns with regards to both healthy and sustainable dietary considerations have never been reported. We report, for the first time, adherence to healthy and sustainable dietary patterns using scoring and regression models, based on recommendations defined by the World Health Organization, in the Dietary Approaches to Stop Hypertension (DASH) study and the EAT-Lancet report, as well as dietary quality based on Dietary Inflammatory Index (DII) among the Hungarian Roma (HR) population living in North East Hungary, with Hungarian general (HG) adults as reference. Data were obtained from a complex, comparative health survey involving dietary assessment, structured questionnaire-based interview, physical and laboratory examinations on 359 HG and 344 HR subjects in Northeast Hungary. Poisson regressions were fit to models that included DASH, EAT, DII and Healthy Diet Indicator as dependent variables to assess the influence of ethnicity on healthy and sustainable nutrient-based patterns. Adjusted models controlled for all relevant covariates using the residual method indicated poor dietary quality with regards to the selected dietary patterns. These associations were not ethnicity-sensitive, except for DII, where Roma ethnicity was linked to a decrease of DII score (β = −0.455, 95%CI: −0.720; −0.191, *p* < 0.05). Currently, HR dietary patterns appear to be relatively unhealthy and unsustainable, rendering them vulnerable to elevated risk of ill-health. Nevertheless, their dietary patterns did not strongly differ from HG, which may contribute to Hungarians being one of the most obese and malnourished nations in Europe. Further prospective research on the potential public and environmental health effects of these findings is warranted.

## 1. Introduction

Ethnicity-based health disparities have been central to the public health discourse in the past decades [1,2,3] and have remained so, particularly amid the current COVID-19 pandemic [4,5,6,7]. Major risk factors for hospitalization, severity and mortality of COVID-19 include diet-related conditions, such as obesity, hypertension and type 2 diabetes, which have been shown to disproportionally affect the most vulnerable [8,9,10]. 

Mounting evidence has consistently shown a strong link between ethnic and socio-economic disparities with dietary and nutrient patterns [11,12,13]. Such inequities exist in many forms, including in diet composition, nutritional behaviors, intake patterns, etc. often resulting in substandard dietary quality, poorer health outcomes and disproportionate burden of disease [14,15]. Along with the growing burden of diet-related noncommunicable diseases (NCDs), there is also a growing interest in characterizing their association with dietary and nutrient patterns in specific, particularly among disadvantaged minority population groups.

In Europe, the Roma population constitutes the largest ethnic minority (estimated to be between 10–12 million) [16] and has been a target of ethnicity-based studies over the past decade [17,18]. Results from different European countries, characterizing dietary aspects of Roma, have revealed inadequate presence of fruits, vegetables [19,20,21,22,23,24,25,26,27,28,29,30,31,32] and dairy products [23,25,26,27,33] in their diet, frequent fast-foods consumption [34,35,36,37], as well as high intake of animal fats [28,31,35,38], sugar-sweetened beverages [22,23,28,37] and sweets [28]. In our previous work, we reported a suboptimal dietary profile and nutritional status of Hungarian Roma (HR) living in segregated colonies in northeastern Hungary, with inadequate nutrient composition and anthropometric status estimates, not strongly different than Hungarian general (HG) population, but occasionally worse among HR. Our findings indicated small differences in meeting nutrient-based dietary recommendations between the two populations, with Roma being less likely to comply with health-promoting nutrient targets [39]. Such information is very useful in informing public health nutrition preventive interventions among Roma, i.e., identifying effective ways of intervening to reduce health inequalities. However, further information on dietary patterns is necessary, in order to gain a higher-resolution and deeper understanding on the current dietary situation and its relation, not only with health, but sustainability considerations as well. What we are eating and how we are producing food is also exerting huge environmental pressures, besides health and nutritional concerns [40]. Diet has emerged as one of the most promising levers to improve health and environmental sustainability, particularly on the demand side of the challenge [41]. It has been demonstrated how health-promoting dietary patterns often have lower environmental impacts, suggesting that dietary shifts that might reduce the risk of NCDs, have the potential to also support attainment of environmental sustainability targets [42,43]. To address the needs emerging from a growing global population, a healthy diet from sustainable food systems was defined by the EAT-Lancet report, a universal reference diet which aims to promote both human health and environmental sustainability [43].

It is reasonable to suppose that diversity and inclusion is key to unlocking sustainability and in creating the kind of development which meets the needs of current generations without compromising the ability of future generations to meet their own needs. Therefore, it is timely and highly relevant to address malnutrition in all its forms and its implications not only with regards to health, but environmental sustainability considerations as well, among underserved groups. With that in mind, we attempt to elucidate the association of Roma ethnicity with regards to dietary patterns shown to strongly influence health (i.e., Healthy Diet Indicator (HDI) and Dietary Inflammatory Index (DII)) and environmental sustainability (i.e., Dietary Approaches to Stop Hypertension (DASH), EAT-Lancet), while considering HG population as reference. To the best of our knowledge, this is the first study among Roma, to date, addressing the relevance of diet for human and planetary health.

## 2. Materials and Methods

### 2.1. Study Design

For this report, we analyzed data obtained from a three-pillar (questionnaire-based, physical examination and laboratory examination) complex (health behavior and examination) survey. Details of sampling and data collection and management are thoroughly described elsewhere [44]. In brief, individuals aged 20 to 64 years, were selected randomly, to be representative of the adult HR population living in segregated colonies of northeast Hungary (Hajdú-Bihar and Szabolcs-Szatmár-Bereg counties), where a great proportion of the HR population resides, as well as that of the HG population living in the same counties. In addition to the demographic, anthropometric, health behavior, physical and laboratory data collection, two 24 h recalls were also obtained to quantify dietary intake. The intended sample size was 500 participants for both study groups, but the final study sample, with full recall data, included 797 participants, of whom 410 subjects were of the HG and 387 individuals of the HR population. The current analysis included 703 participants (359 HG and 344 HR). Detailed information on design, sampling, piloting and validation of the dietary instrument are described elsewhere [39]. In brief, dietary intake data were collected in the case of each participant through a double (one non-consecutive weekday and a weekend day), interviewer-assisted, multiple-pass 24 h dietary recall protocol developed and validated in our previous study [45].

### 2.2. Dietary Patterns Indexes

In this analysis we used four different nutrient-based dietary quality indexes: HDI, DII, the EAT-Lancet and DASH. EAT-Lancet and DASH are considered environmentally sustainable dietary regimes, in addition to being beneficial to health [46]. The World Health Organization (WHO) guidelines for the prevention of chronic diseases [47] and the 2020 updated healthy diet fact sheet [48] were used to construct a modified version of the HDI originally introduced by Huijbregts et al. [49]. Our HDI used seven nutrient standards and a dichotomous variable was generated for each nutrient according to supplementary Table S1 coding criteria. Further, individual scores were summed and participants received a maximum HDI score of 7 points, if all HDI targets were met, and a minimum of 0 points if none was met. Categories of adherence were created based on the total score (i.e., very low HDI (0–1 points), low HDI (2,3), moderate HDI (4,5), high HDI (6,7)).

Our DASH diet index, used previously by Mellen et al. [50], was an entirely nutrient-based version, constructed on the basis of target nutrient values from the DASH diet used in 2 clinical trials [51,52]. The nine nutrients were those expected to be higher (protein, fiber, magnesium, calcium, and potassium) or lower (total fat, saturated fatty acids (SFAs), sodium, and cholesterol). Individual dietary data were compared against each of the nine dietary component goals. Meeting a dietary component goal received 1 point, while meeting an intermediate goal (defined as the midpoint between the DASH diet goal and the nutrient content of the DASH control diet [51]) received 0.5 point, and meeting neither goal received 0 points. The total score was generated by summing all nine nutrient targets score for a minimum of 0 and a maximum of 9 points (see supplementary Table S2). Further, a categorical outcome was evaluated to estimate the number of individuals achieving modest accordance with the DASH dietary approach. Individuals meeting at least half of the DASH targets (DASH score ≥4.5) were considered DASH accordant and the rest, DASH non-accordant.

In 2019, the EAT-Lancet Commission on Healthy Diets from the Sustainable Food Systems report, defined a universal reference diet to promote human and planetary health [43]. To evaluate the adherence of our subjects to this diet, we constructed a novel, nutrient-based EAT index (NB-EAT), based on the nutrient composition of the original EAT-Lancet reference diet. Our NB-EAT included twelve nutrient reference intakes (i.e., α-linolenic acid, carbohydrates, cholesterol, dietary fibers, mono- and poly-unsaturated fats, proteins, saturated fats, total fat, calcium, added sugar, magnesium and potassium) [43].

The individual NB-EAT score was calculated as the sum of nutrient targets met: a value of 1 was given if the participant achieved an EAT-Lancet target for a nutrient and a value of zero was given if target was not met (with a score range from 0 to 12). Categories of adherence were coded based on the achieved score and three categories were created, i.e., low (0–4), moderate (5–8) and high (>8) adherence (supplementary Table S3).

Calculations of dietary inflammatory index (DII) were based on a protocol described by Shivappa et al. [53], while considering methodological aspects for its use and utility [54]. Briefly, nutrient data obtained by 2-day 24 h dietary recall were first linked to the regionally representative world database that was created at the University of South Carolina and provided a robust estimate of a mean and standard deviation for each parameter [53]. These became the multipliers used to express an individual’s exposure, relative to the “standard global mean” as a z-score. This was achieved by subtracting the “standard mean” from the amount reported and then dividing this value by the “standard deviation”. To minimize the effect of “right skewing”, this value was converted to a centered percentile score. The centered percentile score for each food parameter was then multiplied by the respective food parameter effect score. This was derived from the structured review and scoring of 1943 qualifying research articles, to obtain a food parameter-specific DII score for the individual. All of the food parameter–specific DII scores were then summed to create the overall DII score for every participant in the study [53]. Out of 45 possible parameters, a total of 27 nutrients were available to calculate the DII for our study population. The overall index obtained by summing the 27 dietary parameters (i.e., total energy, carbohydrate, protein, total fat, alcohol, fiber, cholesterol, SFAs, caffeine, monounsaturated fatty acids (MUFAs), polyunsaturated fatty acids (PUFAs), omega-3 and omega-6 fatty acids, niacin, thiamine, riboflavin, vitamin B12, vitamin B6, iron, magnesium, zinc, vitamin A, vitamin C, vitamin D, vitamin E, folic acid and beta-carotene), ranged from a minimum of −4.60 (most anti-inflammatory) to a maximum of 3.12 points, with a median value of −1.33. Since there is no current consensus on the optimal DII score values, we addressed this challenge by creating tertiles of the DII score, i.e., statistically dividing our DII score distribution into three parts (=three quantiles).

### 2.3. Data Analyses

Descriptive univariate analysis with chi-square test were used to evaluate the association between DASH, HDI, DII and NB-EAT scores, as well as socio-demographic and anthropometric factors such as age, sex, and BMI. We considered negative binomial and Poisson regressions for exploring differences between the HG and HR populations with regards to nutrient patterns. Negative binomial regression was chosen if data showed over-dispersion. All sociodemographic and nutritional covariates (age, sex, education, marital status, perceived financial situation, economic activity, BMI and energy intake) were included in the models in a block manner to determine the Roma ethnicity effect, independently from sociodemographic and anthropometric data. Initially, regression models were fit with DASH, HDI, DII and NB-EAT scores as outcome variables and Roma ethnicity controlled for anthropometric data. Secondly, demographic variables were added to each model. Finally, the regression models were fitted with demographic, anthropometric and socioeconomic factors together. Complete regression analysis results and models are provided in supplementary Tables S4–S7. Statistical analyses were carried out using SPSS 26.0 (SPSS, version 26.0; IBM Corp., Armonk, NY, USA) software and R Statistics/R Studio. This work has reported results in accordance with STROBE (STrengthening the Reporting of OBservational studies in Epidemiology) extension for nutrition and dietary assessment [55].

### 2.4. Research Ethics

Approval for the research protocols and methodology was provided by the Ethical Committee of the Hungarian Scientific Council on Health (61327-2017/EKU). Participants gave their written informed consent in both study populations in accordance with the Declaration of Helsinki and the Science Ethics Code of The Hungarian Academy of Sciences.

## 3. Results

Table 1 shows relevant basic and nutritional characteristics of our samples. There were no strong statistical differences between the two groups, in terms of basic characteristics and energy intake. Regarding nutrient intake there were significant differences for sodium and cholesterol, both which were higher among HG. Sugar and total carbohydrate (both as percentage of total energy) intakes were significantly higher among HR. Significant associations were observed between ethnicity and economic activity, educational level and perceived financial wellbeing, but there was no association between ethnicity and marital status.

### Dietary Pattern Scores and Quality

Further, when accounting for dietary patterns quality, results showed a high representation of participants with poorer adherence levels for DASH, HDI and NB-EAT, independently of the dietary index used, ethnicity or sex (Table 2). DII tertile and score distribution also showed a considerable representation in the two upper tertiles. Additionally, there was no observed statistical association between sex/ethnicity and the selected dietary indexes, with regards to score differences.

Multivariable regression models (Table 3), both adjusted and unadjusted for the indicated and relevant covariates, showed no significant effect of Roma ethnicity on DASH, NB-EAT and HDI scores. On the other hand, DII score was significantly and inversely associated with Roma ethnicity in the adjusted models.

## 4. Discussion

Our results indicate substandard adherence to established healthy and sustainable dietary guidelines, as accounted by the nutrient-based dietary indexes used in this work. Ethnicity did not have a strong influence on adherence to selected dietary guidelines. However, being Roma was associated with a lower DII score, i.e., lower dietary inflammatory potential. These findings are in line with our previous results and reinforce the fact that currently the Hungarian population is not close to meeting healthy diet targets, regardless of ethnic background. The cause of such a substandard quality of diet is highly likely to be multifactorial. A relevant contributor may be the lack of adequate dietary guidance/interventions, as nutrition services have not yet been mainstreamed into the Hungarian health care system.

As a result, dietary patterns such as DASH, EAT-Lancet or dietary evaluation based on DII and HDI approach have not been widely promoted in Hungary. At present, provision of general preventive services in primary health care in Hungary is challenging and not based on evidence-informed dietary guidance, i.e., trained dieticians, nutritional experts, etc. [56].

General practitioners (GPs) constitute a significant component of the Hungarian primary healthcare system and are in regular contact with both the healthy and ailing people. According to our data and previous research, lifestyle counselling is the kind of service that patients need most and one of the leading drivers of medical litigation [57]. Coverage and quality of nutritional counselling services, within primary healthcare settings are limited and primarily focused on those suffering from diet-related NCDs, particularly among type 2 diabetes patients [58,59]. People suffering from other diet-related NCDs (e.g., Crohn’s disease, gluten sensitivity, etc.) are provided with some dietary advice, only within the context of outpatient care, with no targeted or tailored preventive dietary counselling yet. Additionally, dietary assessment is not common in the routine GP’s practice and it is provided only for small proportion of patients (i.e., 24% and mainly hypertonic and diabetic), with almost no monitoring on compliance or providing further counselling and/or follow-up [60].

Dietary services, currently available only in the outpatient and inpatient care, appear to target only those who are already suffering from diet-related NCDs, hence not as a preventive approach. On the other hand, there is a limited number of private dietary and nutritional services, available only in the largest cities, relatively pricy and not clear whether they are effective or not. However, it is crucial to recognize that optimal health and well-being is a human right and not a privilege of only those who can afford to pay. Integrating dietary services within health systems has the potential to generate substantial health gains, while simultaneously being cost-effective [61].

Another aspect of the inadequate access and/or availability of evidence-based nutritional services that merits attention, is the need to raise the profile of nutrition at national level, while aligning resource allocation accordingly. Currently, there is a demand for such services, particularly among high-income, highly-educated Hungarians [62], that seem to have recognized the value of nutritional guidance and interventions (e.g., balanced nutrition, blood glucose lowering and healthy weight control, etc.), among other lifestyle changes.

Currently, nutrition services are not widely supported by the Hungarian national health budget and are typically not delivered by qualified nutrition professionals, since other professions (e.g., personal trainers, self-proclaimed dietitians, etc.) are currently attempting to fill the demand gap [63]. The latter phenomenon has been recognized and there is data showing that such guidance is inadequate and not in line with established guidelines [63,64]. Consequently, despite efforts to improve nutrition [65], Hungarians appear to have a limited exposure to professional and evidence-informed dietary and nutritional preventive services, something which may contribute to our “no difference” findings with regards to nutrient-based dietary patterns.

Additionally, there was a considerable representation of subjects in the upper tertile of the DII in our sample, hinting an elevated inflammatory potential of current dietary patterns. Chronic inflammation plays an important role in the development of several chronic diseases [66].

Since various nutrients and foods have been shown to modulate inflammation, dietary patterns play an important role in the regulation of chronic inflammation [67]. Although the link between diet and disease outcomes needs additional studies to further confirm the health potential of current dietary patterns, longitudinal epidemiological data have already linked poor adherence to healthy dietary patterns to many NCDs and claiming an attributable global death toll of 11 million from diet-related NCDs [68].

Therefore, there is a compelling case for urgently considering the inclusion of nutrition and dietary services as an integral component of primary healthcare [69]. The Hungarian healthcare system has for decades focused on the clinical, pharmacological-oriented model of disease that may ignore fundamental modifiable causes, such as diet and lifestyle. The consequences of this approach can be observed in the poor dietary patterns reported here, with the potential to contribute to an elevated risk of diet-related NCDs. This is further supported by data showing a very high prevalence of metabolic syndrome in both HG and HR populations (i.e., 39.8% and 44.0%, respectively), with no significant difference between the two groups in either females or males [44]. Integrating and mainstreaming nutrition actions into the Hungarian health care system to promote healthier diet, and prevent and treat diet-related NCDs, has the potential to generate substantial health gains and can be highly cost-effective [61].

Furthermore, adherence to sustainable dietary patterns among our participants, can be viewed, not only as a dietary marker, but as one of behavioral commitment towards addressing Climate Change as well. The vast majority of nutrient-based EAT-Lancet reference diet targets were not attained and none of the participants was in the third-upper category of adherence. Considering the detrimental environmental impact of current food systems [70], and concerns raised about their sustainability, there is a pressing need to promote diets that are healthy and have no or low destructive impact on the environment in Hungary and globally. At present, the ‘Nutritional recommendations for the adult population in Hungary’ (i.e., national food-based dietary guideline [71]) fails to include sustainability criteria, although there is mounting evidence linking overconsumption of, in particular, red and ultra-processed meat products with detrimental human and environmental health outcomes [42,72,73]. Advocating for plant-based diets in Hungary is also timely. Recommending dietary shifts towards plant-based diets may be of great importance in achieving health and sustainability goals [74], as from a food systems point of view, down-right adoption of plant-based diets has the potential to simultaneously optimize food supply, improve health, increase environmental sustainability and advance social justice outcomes [75,76]. 

Apart from the established health benefits [77,78,79] DASH diet is also considered an environmental-friendly dietary pattern [46,80]. Our results indicate an extremely high “non-accordance” to DASH pattern (95%), independently of ethnicity. This may be an epidemiological signature, which may signify increased risk for diet-related NCDs, as well as a low potential of the current diet to contribute in improving climate targets. Thus, our findings provide novel insights into dietary situation among HR and HG, as well as key dietary recommendations, which might require special attention during nutrition/public health education campaigns. Moreover, we advocate for nutrition education and research to be extensively integrated in health sciences-related academic programs, with an overarching emphasis and regular reinforcement of the importance of higher fiber, fruit, vegetable and wholegrain intake and substitution of fat sources with beneficial ones, in an energy balanced manner.

In addition to the above-mentioned challenges, the actual nutrition situation is clearly neither a mere consequence of inappropriate quantity/quality of foods in the Hungarian diet, nor as a lack of willpower from the individual [81], but as a consequence of a fundamental global challenge: food systems that have failed in providing healthy, safe, affordable and sustainable diets [82].

The economic, social and environmental implications of further inaction can impact the growth and development of individuals and societies for decades to come [83,84]. As the Lancet Series on the “Double Burden of Malnutrition” has shown, the intricate biological and social pathways of all forms of malnutrition cannot be disrupted through siloed interventions, therefore requiring society-wide and scalable behavioral shifts that can be sustained over time [85,86]. Our findings also point towards the need for triple-duty actions, as described by Swinburn et al. [41] as The Lancet Commission report. Such policy actions have the potential to re-orient major systems of food and agriculture, transport, urban design, and land use that drive this Syndemic—the three co-existing pandemics: obesity, undernutrition and climate change. Such actions need to occur locally, nationally and within a global framework. Implementation of such actions to address these deeper drivers is politically more difficult to achieve and their outcomes are more uncertain compared to downstream actions such as health promotion programs or healthcare service provision. However, their implementation is essential for transformative, systemic changes. More studies are warranted to determine the food system determinants, social drivers of poor dietary intake in Hungary, as well as trials investigating how dietary interventions may effectively influence the current dietary patterns, particularly among Roma.

Finally, the current COVID-19 pandemic has cast a spotlight on longstanding costly and life-threatening inequities that exist in our global society. Those living in economically challenged communities, such as ethnic minorities, are bearing the heaviest burden of COVID-19 infections. It is now accepted that poor metabolic health is one of the most important immunity-impairing factors underlying cardiovascular disease, type 2 diabetes and obesity-related cancers, rendering many people vulnerable to COVID-19 severity and mortality [87]. However, while diet-related NCDs may increase vulnerability to the virus, limited attention has been paid in improving access to healthy and sustainable diets, that can both sustain metabolic health, support a vigorous immune system and contribute to lessening the effect of our footprint on the planet. After this pandemic subsides, a lot more attention needs to be given to the power that our diets have to ward off, not only future medical, economic and social calamities from whatever pathogen next comes down the pike, but to address the bigger “pandemic” as well: climate change.

As governments embark on economic recovery plans in the wake of COVID-19, a great opportunity exists, within the framework of the UN Decade of Action on Nutrition (2016–2025), to invest in a green recovery plan that can tackle the health equity and environmental crises together, to ensure the most effective response to each. Addressing these issues and building forward better starts with our “plates”.

### Limitations, Strengths and Future Outlooks

There are some potential limitations to our observations that should be recognized. This analysis is based on a double 24 h dietary recall, hence findings need to be interpreted with caution, as long-term dietary patterns of the population under investigation may not be fully captured by this approach. Further, the current findings, although insightful, are relatively incomplete, as linking them with health outcome data (e.g., metabolic syndrome) is crucial in order to better characterize the current situation, and link health effects of an environmentally sustainable diet and further confirm these findings. Although diet is, no doubt, an important modulator of inflammation, it is by no means the only one. Other indexes, including physical activity and stress, should be derived using similar methods. If these could be integrated with the DII, then this could validate and confirm the inflammatory potential of the diet in the current population under investigation. It should be taken also into consideration that, in our study, the representation of females among HR was higher than among HG. 

This has also been the case in our previous surveys conducted among segregated Roma colonies in Hungary [88] and also in Roma surveys in other countries [89]. Other potential limitations related to the composition of our sample are described in detail in our previous work [39]. Although this is the first attempt, to the best of our knowledge, to present a nutrient-based index for healthy and sustainable diets based on the rigorous EAT-Lancet reference diet, we recognize that it may need further validation. The EAT-Lancet commission’s “healthy and sustainable reference diet” provides values of nutrient composition, as well as intakes for food groups, with the latter being more informative. In our attempt to describe dietary sustainability, NB-EAT’s use was chosen due to the inability to obtain dietary intake data at the level of individual food items or food group data—something which we acknowledge that can provide more tangible details on the healthiness and sustainability aspects of the diet. Nevertheless, it was shown that there is a poor adherence to healthy and sustainable dietary targets, independently of the dietary pattern and population group.

Further work on drivers of poor dietary patterns should go beyond measuring the effect of prescribed (but often not followed) dietary guidelines on population-averaged cohorts, towards quantifying the efficiency of dietary and lifestyle advice as well. Even the best dietary advice in the world may be indistinguishable from the worst, when individuals do not or cannot adhere to it due to specific circumstances, e.g., place of residence, access to healthy foods, employment conditions and income. Despite the abovementioned challenges, our findings offer new nutritional insights on dietary aspects that require particular attention during potential interventions and monitoring their effects, when attempting to improve the overall quality of the diet among young adults in Hungary. Decision-makers and experts should approach this issue from a food system’s perspective, in order to address and transform the complex web of activities involving the production, processing, transport and consumption of unhealthy diets.

## 5. Conclusions

Potential nutritional interventions in Hungary, addressing healthy and sustainable nutrition, are not only necessary among Hungarian Roma population, but on a population-wide level as well. Unhealthy nutrient-based dietary patterns appear generally indiscriminate of ethnic background according to our analyses, with both populations (HG and HR) poorly adhering to healthy and sustainable dietary patterns, with no strong mediation by any included factor in this analysis. Identifying dietary patterns that are nutrient-rich, affordable, healthy and sustainable for Hungarians should be a top public health research priority, as well as an opportunity to discern and address social inequalities in nutrition and health. Our cross-sectional analysis also indicates that current nutritional trajectory may not be in line with achieving the sustainable development goals in respect to multiple dietary targets for public health and environmental sustainability. Research and policy action therefore need to be integrated between disciplines and domains, starting with the formulation of integrated national nutrition guidelines, through a sustainability lens and an outlook of long-term challenges to the food system, as well as an agenda to achieve the relevant behavioral change. Further research is warranted to elucidate drivers and ascertain food-based dietary patterns, with sustainability considerations in mind.

## Figures and Tables

**Table 1:** Characteristics of study samples.

Characteristics		Variable ^†^	Hungarian General (*n* = 344)	Hungarian Roma (*n* = 359)	*p* ^‡^
Basic characteristics	Demographics	Age (years)—mean (std. dev.)	44 (12)	43 (12)	NS
		Females—*n* (%)	188 (52.4)	**248 (72.1)**	***
	Education	Elementary—*n* (%)	76 (21.2)	**292 (84.9)**	***
		Secondary/Vocational education—*n* (%)	230 (64.1)	**52 (15.1)**	
		University degree or higher—*n* (%)	53 (14.8)	**0 (0)**	
	Economic activity	Some type of full-/part-time employment—*n* (%)	296 (82.5)	**256 (74.4)**	**
		Student—*n* (%)	8 (2.2)	**0 (0)**	
		Unable to work/Retired—*n* (%)	40 (11.1)	**32 (9.3)**	
		Unemployed—*n* (%)	15 (4.2)	**56 (16.3)**	
	Marital status	Unmarried/Divorced/Widowed—*n* (%)	136 (37.9)	113 (32.8)	NS
		Married—*n* (%)	223 (62.1)	231 (67.2)	
	Perceived financial wellbeing	Good or very good—*n* (%)	115 (32.8)	**51 (15.0)**	***
		Fair—*n* (%)	190 (54.1)	**186 (54.7)**	
		Challenging or very challenging—*n* (%)	46 (13.1)	**103 (30.3)**	
Nutritional characteristics	Energy intake	Energy (kJ/day)	9146.9 (8824.7; 9469.1)	8836.9 (8537.0; 9136.8)	NS
		Energy (kcal/day)	2188.3 (2111.2; 2265.3)	2114.1 (2042.4; 2185.8)	NS
	Anthropometrics	BMI (kg/m^2^)	27.26 (26.7; 27.8)	27.66 (27.0; 28.4)	NS
	Macronutrient intake	Fiber (%E)	3.9 (3.7; 4.0)	4.0 (3.8; 4.2)	NS
		Fiber (g/1000 kcal)	9.7 (9.2; 10.1)	9.9 (9.4; 10.4)	NS
		Protein (%E)	**15.6 (15.2; 15.9)**	15.1 (14.7; 15.4)	*
		Fat (%E)	37.2 (36.3; 38.0)	36.1 (35.2; 37.0)	NS
		SFA (%E)	10.7 (10.3; 11.1)	10.66 (10.3; 11.0)	NS
		Carbohydrates (%E)	46.2 (45.3; 47.1)	**48.2 (47.2; 49.2)**	**
		Sugar (g)	96.3 (89.0; 103.5)	101.5 (94.1; 108.8)	NS
		Sugar (%E)	17.0 (16.0; 18.0)	**18.8(17.7; 19.8)**	*
		UFA (g)	**20.9 (20.4; 21.4)**	19.7 (19.1; 20.2)	**
		Cholesterol (mg/1000 kcal)	**172.9 (164.7; 181.0)**	159.5 (152.2; 166.8)	*
	Micronutrient intake	Magnesium (mg/1000 kcal)	193.1 (166.0; 220.2)	181.6 (173.8; 189.5)	NS
		Calcium (mg/1000 kcal)	248.5 (233.51; 263.49)	248.0 (234.4; 261.7)	NS
		Potassium (mg/1000 kcal)	1391.1 (1311.4; 1470.8)	1435.3 (1353.3; 1517.4)	NS
		Sodium (mg/1000 kcal)	**2628.1 (2522.8; 2733.4)**	2458.3 (2365.7; 2550.8)	*

^†^ All variable values are given with their respective 95% CI, unless otherwise indicated. ^‡^ * *p* ≤ 0.05, ** *p* ≤ 0.01, *** *p* ≤ 0.001: T-test or Mann–Whitney for differences and Chi-square for associations. Significantly higher values are bolded where appropriate. SFA: saturated fatty acids; UFA: unsaturated fatty acids; (%E): intake as percentage of total energy; IQR: interquartile range. Note: For “Perceived financial wellbeing” some responses were missing.

**Table 2:** Distribution of the dietary indicators among Hungarian general and Roma by sex.

Dietary Indicator		Hungarian General			Hungarian Roma		
		Both Sexes (A)	Females (C)	Males (E)	Both Sexes (B)	Females (D)	Males (F)
HDI	Very low	27 (7.5)	13 (6.9)	14 (8.2)	23 (6.7)	18 (7.3)	5 (5.2)
	Low	172 (47.9)	91 (48.6)	81 (47.4)	152 (44.2)	113 (45.6)	39 (40.6)
	Moderate	140 (39.0)	75 (39.9)	65 (37.0)	158 (45.9)	111 (44.7)	47 (48.9)
	High	20 (5.6)	9 (4.6)	11 (6.4)	11 (3.2)	6 (2.4)	5 (5.2)
DASH	Non-accordant	341 (95.0)	177 (94.1)	164 (95.9)	330 (95.9)	240 (96.8)	90 (93.8)
	Accordant	18 (5.0)	11 (5.9)	7 (4.1)	14 (4.1)	8 (3.2)	6 (6.2)
NB-EAT	Low	324 (90.3)	171 (91.0)	153 (89.5)	305 (88.7)	223 (89.9)	82 (85.4)
	Moderate	35 (9.7)	17 (9.0)	18 (10.5)	39 (11.3)	25 (10.1)	14 (14.6)
	High	0 (0.0)	0 (0.0)	0 (0.0)	0 (0.0)	0 (0.0)	0 (0.0)
DII	Tertile 1	119 (33.1)	66 (35.1)	53 (31.0)	115 (33.5)	84 (33.9)	31 (32.3)
	Tertile 2	109 (30.4)	50 (26.6)	59 (34.5)	126 (36.6)	90 (36.3)	36 (37.5)
	Tertile 3	131 (36.5)	72 (38.3)	59 (34.5)	103 (29.9)	74 (29.8)	29 (30.2)
DASH score (0–9): median (IQR)		1.5 (1.5; 2.0)	1.5 (1.5; 2.0)	1.5 (1.5; 2.0)	1.5 (1.5; 2.0)	1.5 (1.5; 2.0)	1.5 (1.5; 2.0)
NB-EAT score (0–12): median (IQR)		2.0 (2.0; 3.0)	2.0 (2.0; 3.0)	2.0 (2.0; 3.0)	2.0 (2.0; 3.0)	2.0 (2.0; 3.0)	3.0 (3.0; 4.0)
HDI score (0–7): median (IQR)		3.0 (3.0; 4.0)	3.0 (3.0; 4.0)	3.0 (3.0; 4.0)	3.0 (3.0; 4.0)	3.0 (3.0; 4.0)	4.0 (4.0; 5.0)
DII score (−4.60–3.12): median (IQR)		−1.26 (−1.49; −1.06)	−1.31 (−1.80; −0.92)	−1.26 (−1.45; −1.02)	−1.40 (−1.65; −1.23)	−1.34 (−1.64; −1.12)	−1.59 (−1.92; −1.27)

All data are given as *n* (%) unless otherwise indicated. IQR: Interquartile Range.

**Table 3:** Effect of Roma ethnicity on nutrient-based dietary patterns *.

Dietary Score	MODEL 1 (β [95%CI])	MODEL 2 (β [95%CI])	MODEL 3 (β [95%CI])
**DASH ^†^**	−0.023 [−0.176; 0.129]	−0.084 [−0.286; 0.117]	−0.049 [−0.254; 0.156]
**HDI ^†^**	0.038 [−0.131; 0.207]	−0.003 [−0.229; 0.223]	−0.001 [−0.231; 0.230]
**DII ^‡^**	−0.147 [−0.344; 0.049]	**−0.450 [−0.709; −0.191]**	**−0.455 [−0.720; −0.191]**
**NB-EAT ^†^**	0.021 [−0.073; 0.114]	−0.024 [−0.183; 0.136]	−0.017 [−0.179; 0.144]

^†^ Poisson regression model; ^‡^ Multiple linear regression model; * Hungarian general population sample is taken as a reference in these models. Model 1: effect adjusted only for BMI and energy intake; Model 2: effect adjusted for BMI, age, education level, energy intake and sex; Model 3: effect adjusted for BMI, age, education level, energy intake, sex, financial status, marital status and economic activity (see supplementary Tables S4–S7 for more information). Significant effect sizes are bolded. DASH: The Dietary Approaches to Stopping Hypertension; HDI: Healthy Diet Indicator; DII: The Dietary Inflammatory Index; EAT: Nutrient-based EAT-Lancet score; β [95%CI]: beta coefficient of the regression model, accompanied by its corresponding 95% confidence interval.

## Data Availability

All relevant data that support the findings of this study are within the paper and its Supplementary materials. Additional data are available from the corresponding author, upon reasonable request.

## Supplementary Materials

The following are available online at https://www.mdpi.com/2072-6643/13/3/721/s1, Table S1. Healthy diet indicator components used in the current study and their coding criteria based on the World Health Organization’s dietary guidelines. Table S2. Nutrient Targets for DASH Score. Table S3. Diet indicator components used in the current study and their coding criteria based on the EAT-Lancet reference diet. Table S4. Regression models for DASH score among Hungarian general and Roma populations. Table S5. Regression models for HDI score among Hungarian general and Roma subjects. Table S6. Regression models for DII score among Hungarian general and Roma subjects. Table S7. Regression models for nutrient-based EAT-Lancet score among Hungarian general and Roma subjects.
