# Supplementary material for: Deteriorated Dietary Patterns with Regards to Health and Environmental Sustainability among Hungarian Roma Are Not Differentiated from Those of the General Population
Source: Nutrients. 2021 Feb 24;13(3):721. doi: 10.3390/nu13030721 (PMC7996132; doi:10.3390/nu13030721)
Supplement: Supplementary file 1 [file nutrients-13-00721-s001.pdf]

Contents

Table S1. Healthy diet indicator components used in the current study and their coding criteria based on the World Health Organization's dietary guidelines [1] 2

Table S2. Nutrient Targets for DASH Score [3] 2

Table S3. Diet indicator components used in the current study and their coding criteria based on the EAT-Lancet reference diet [2] 2

Table S4. Regression models for DASH score among Hungarian general and Roma populations 3

Table S5. Regression models for HDI score among Hungarian general and Roma subjects 3

Table S6. Regression models for DII score among Hungarian general and Roma subjects 3

Table S7. Regression models for nutrient-based EAT-Lancet score among Hungarian general and Roma subjects 4

References 4

Table S1. Healthy diet indicator components used in the current study and their coding criteria based on the World Health Organization's dietary guidelines [1]

No.	Nutrient or food group (*daily intake*)	Dichotomous value	
		1	0
1	Saturated fatty acids (%*E*)	0-10	>10
2	Polyunsaturated fatty acids (%*E*)	6-10	<6 or >10
3	Protein (%*E*)	10-15	<10 or >15
4	Total dietary fibre (*g*)	>25	<25
5	Monosaccharides and disaccharides (%*E*)	0-10	>10
6	Cholesterol (*mg*)	0-300	>300
7	Potassium (*mg*)	≥3500	<3500

E%: Nutrient given as percentage of total daily energy; *g*: grams; *mg*: milligrams.

Table S2. Nutrient Targets for DASH Score [3]

Nutrient	DASH Diet Nutrient Composition	DASH Target	Intermediate Target
Saturated fats	6%E	6%E	11%E
Total fat	27%E	27%E	32%E
Protein	18%E	18%E	16.5%E
Cholesterol	150 mg	71.4 mg/1000 kcal	107.1 mg/1000 kcal
Fibre	31 g	14.8 g/1000 kcal	9.5 g/1000 kcal
Magnesium	500 mg	238 mg/1000 kcal	158 mg/1000 kcal
Calcium	1240 mg	590 mg/1000 kcal	402 mg/1000 kcal
Potassium	4700 mg	2238 mg/1000 kcal	1534 mg/1000 kcal
Sodium	2400 mg	1143 mg/1000 kcal	1286 mg/1000 kcal

[a] Based on a 2100-kcal diet. Abbreviation: DASH, Dietary Approaches to Stop Hypertension trial [4]. *E%:* Nutrient given as percentage of total daily energy; *g*: grams; *mg*: milligrams.

Table S3. Diet indicator components used in the current study and their coding criteria based on the EAT-Lancet reference diet [2]

Dietary element	Reference amount	E%*
Alpha linolenic acid (g)	≥2.5	≥0.9
Carbohydrates (g)	≥317.3	≥50.8
Cholesterol (mg)	≤125.2	≤50.1 (mg/1000 kcal)
Dietary fibres (g)	≥42.9	≥6.9
Mono- and poly-unsaturated fats (g)	≥75.9	≥27.3
Proteins (g)	90.1	14.4
Saturated fats (g)	≤22.7	≤8.2
Total fat (g)	≤105.6	≤38.0
Calcium (mg)	≥717.8	≥287.1 (mg/1000 kcal)
Magnesium (mg)	≥732.5	≥293.0 (mg/1000 kcal)
Potassium (mg)	≥4100.7	≥1640.3 (mg/1000 kcal)
Added sugar (g)	≤31.0	5.0

**Dietary element expressed as percentage of total energy, unless otherwise indicated. g*: grams; *mg*: milligrams.

Table S4. Regression models for DASH score among Hungarian general and Roma populations

DASH†	MODEL 1 *β [95%CI]*	MODEL 2 *β [95%CI]*	MODEL 3 *β [95%CI]*
Roma	-0.023 [-0.176 ; 0.129]	-0.084 [-0.286 ; 0.117]	-0.049 [-0.254 ; 0.156]
BMI (kg/m^2)	0.004 [-0.008 ; 0.016]	0.005 [-0.008 ; 0.018]	0.005 [-0.008 ; 0.018]
Energy intake (kcal)	**0.000 [0.000 ; 0.000]**	**0.000 [0.000 ; 0.000]**	**0.000 [0.000 ; 0.000]**
Age (years)		0.000 [-0.007 ; 0.006]	0.000 [-0.008 ; 0.007]
Secondary/Vocational education ref: Elementary		-0.105 [-0.307 ; 0.096]	-0.105 [-0.306 ; 0.095]
University degree or higher ref: Elementary		0.037 [-0.298 ; 0.373]	0.043 [-0.301 ; 0.386]
Females ref: males		0.093 [-0.068 ; 0.254]	0.074 [-0.089 ; 0.237]
Financial status: good. ref: challenging			0.144 [-0.098 ; 0.387]
Financial status: fair. ref: challenging			0.194 [-0.003 ; 0.392]
Economic activity: employed. ref: unemployed			0.084 [-0.178 ; 0.346]
Economic activity: inactive. ref: unemployed			0.155 [-0.206 ; 0.517]
Marital status: coupled. ref: single			0.015 [-0.152 ; 0.183]

† Poisson regression model
**Hungarian general is taken as a reference group in the model.*
***Model 1: effect adjusted only for BMI and energy intake; Model 2: effect adjusted for BMI, age, education level, energy intake and sex; Model 3: effect adjusted for BMI, age, education level, energy intake, sex, financial status, marital status and economic activity. β [95%CI]: beta coefficient of the regression model, accompanied by its corresponding 95% confidence interval.*

Table S5. Regression models for HDI score among Hungarian general and Roma subjects

HDI†	MODEL 1 *β [95%CI]*	MODEL 2 *β [95%CI]*	MODEL 3 *β [95%CI]*
Roma	0.038 [-0.131 ; 0.207]	-0.003 [-0.229 ; 0.223]	-0.001 [-0.231 ; 0.230]
BMI (kg/m^2)	0.004 [-0.010 ; 0.018]	0.004 [-0.010 ; 0.018]	0.004 [-0.010 ; 0.018]
Energy intake (kcal)	**0.000 [0.000 ; 0.000]**	**0.000 [0.000 ; 0.000]**	**0.000 [0.000 ; 0.000]**
Age (years)		-0.001 [-0.008 ; 0.007]	0.001 [-0.007 ; 0.009]
Secondary/Vocational education ref: Elementary		-0.065 [-0.290 ; 0.160]	-0.070 [-0.296 ; 0.156]
University degree or higher ref: Elementary		-0.040 [-0.415 ; 0.334]	-0.044 [-0.426 ; 0.339]
Females ref: males		0.013 [-0.165 ; 0.192]	0.012 [-0.168 ; 0.191]
Financial status: good. ref: challenging			0.067 [-0.201 ; 0.335]
Financial status: fair. ref: challenging			0.107 [-0.110 ; 0.325]
Economic activity: employed. ref: unemployed			-0.012 [-0.301 ; 0.278]
Economic activity: inactive. ref: unemployed			-0.089 [-0.488 ; 0.310]
Marital status: coupled. ref: single			0.012 [-0.171 ; 0.194]

† Poisson regression model
**Hungarian general is taken as a reference group in the model.*
***Model 1: effect adjusted only for BMI and energy intake; Model 2: effect adjusted for BMI, age, education level, energy intake and sex; Model 3: effect adjusted for BMI, age, education level, energy intake, sex, financial status, marital status and economic activity. Significant associations are bolded. HDI:* Healthy Diet Indicator [1,6]. *β [95%CI]: beta coefficient of the regression model, accompanied by its corresponding 95% confidence interval.*

Table S6. Regression models for DII score among Hungarian general and Roma subjects

DII†	MODEL 1 *β [95%CI]*	MODEL 2 *β [95%CI]*	MODEL 3 *β [95%CI]*
Roma	**-0.147 [-0.344 ; 0.049]**	**-0.450 [-0.709 ; -0.191]**	**-0.455 [-0.720 ; -0.191]**
BMI (kg/m^2)	0.005 [-0.011 ; 0.021]	0.006 [-0.010 ; 0.022]	0.005 [-0.011 ; 0.022]
Energy intake (kcal)	**0.001 [0.001 ; 0.001]**	**0.001 [0.001 ; 0.001]**	**0.001 [0.001 ; 0.001]**
Age (years)		0.002 [-0.007 ; 0.010]	0.003 [-0.006 ; 0.012]
Secondary/Vocational education ref: Elementary		-0.502 [-0.760 ; -0.244]	**-0.502 [-0.760 ; -0.244]**
University degree or higher ref: Elementary		-0.121 [-0.551 ; 0.309]	-0.059 [-0.496 ; 0.378]
Females ref: males		0.204 [-0.001 ; 0.409]	0.195 [-0.010 ; 0.401]
Financial status: good. ref: challenging			0.019 [-0.288 ; 0.326]
Financial status: fair. ref: challenging			**0.325 [0.076 ; 0.574]**
Economic activity: employed. ref: unemployed			-0.115 [-0.447 ; 0.218]
Economic activity: inactive. ref: unemployed			-0.196 [-0.649 ; 0.257]
Marital status: coupled. ref: single			0.030 [-0.179 ; 0.239]

† Multiple linear regression model
**Hungarian general is taken as a reference group in the model.*

***Model 1: effect adjusted only for BMI and energy intake; Model 2: effect adjusted for BMI, age, education level, energy intake and sex; Model 3: effect adjusted for BMI, age, education level, energy intake, sex, financial status, marital status and economic activity Significant associations are bolded. DII: The Dietary Inflammatory Index [5]. β [95%CI]: beta coefficient of the regression model, accompanied by its corresponding 95% confidence interval.*

Table S7. Regression models for nutrient-based EAT-Lancet score among Hungarian general and Roma subjects

EAT †	MODEL 1<br>*β [95%CI]*	MODEL 2<br>*β [95%CI]*	MODEL 3<br>*β [95%CI]*
Roma	0.021 [-0.073 ; 0.114]	-0.024 [-0.183 ; 0.136]	-0.017 [-0.179 ; 0.144]
BMI (kg/m^2)	0.005 [-0.019 ; 0.029]	0.003 [-0.021 ; 0.027]	0.006 [-0.019 ; 0.030]
Energy intake (kcal)	**0.000 [0.000 ; 0.000]**	**0.000 [0.000 ; 0.000]**	**0.000 [0.000 ; 0.000]**
BMI (kg/m^2)*Energy intake (kcal)	**0.000 [0.000 ; 0.000**	**0.000 [0.000 ; 0.000]**	**0.000 [0.000 ; 0.000]**
Age (years)		0.002 [-0.002 ; 0.006]	0.003 [-0.001 ; 0.008]
Secondary education ref: Elementary		-0.042 [-0.242 ; 0.157]	-0.065 [-0.266 ; 0.136]
Vocational or higher ref: Elementary		-0.067 [-0.235 ; 0.101]	-0.093 [-0.264 ; 0.078]
Secondary/Vocational education*Roma		0.026 [-0.293 ; 0.344]	0.065 [-0.256 ; 0.386]
Vocational or higher*Roma		0.049 [-0.267 ; 0.364]	0.086 [-0.234 ; 0.407]
Females ref: males		0.018 [-0.082 ; 0.119]	0.019 [-0.082 ; 0.120]
Financial status: good. ref: challenging			0.156 [0.005 ; 0.307]
Financial status: fair. ref: challenging			0.111 [-0.013 ; 0.234]
Economic activity: employed. ref: unemployed			0.036 [-0.129 ; 0.200]
Economic activity: inactive. ref: unemployed			0.019 [-0.204 ; 0.241]
Marital status: coupled. ref :single			0.028 [-0.075 ; 0.130]

† Poisson regression model

**Hungarian general is taken as a reference group in the model.*

***Model 1: effect adjusted only for BMI and energy intake; Model 2: effect adjusted for BMI, age, education level, energy intake and sex; Model 3: effect adjusted for BMI, age, education level, energy intake, sex, financial status, marital status and economic activity Significant associations are bolded. EAT: Nutrient-based EAT-Lancet score [7]; β [95%CI]: beta coefficient of the regression model, accompanied by its corresponding 95% confidence interval.*

References

1. Nishida, C.; Uauy, R.; Kumanyika, S.; Shetty, P. The joint WHO/FAO expert consultation on diet, nutrition and the prevention of chronic diseases: process, product and policy implications. *Public Health Nutr* **2004**, *7*, 245-250, doi:10.1079/phn2003592.
2. Willett, W.; Rockström, J.; Loken, B.; Springmann, M.; Lang, T.; Vermeulen, S.; Garnett, T.; Tilman, D.; DeClerck, F.; Wood, A. Food in the Anthropocene: the EAT–Lancet Commission on healthy diets from sustainable food systems. *Lancet* **2019**, *393*, 447-492, doi:10.1016/S0140-6736(18)31788-4.
3. Mellen, P.B.; Gao, S.K.; Vitolins, M.Z.; Goff, D.C., Jr. Deteriorating Dietary Habits Among Adults With Hypertension: DASH Dietary Accordance, NHANES 1988-1994 and 1999-2004. *JAMA Intern Med* **2008**, *168*, 308-314, doi:10.1001/archinternmed.2007.119.
4. Sacks, F.M.; Obarzanek, E.; Windhauser, M.M.; Svetkey, L.P.; Vollmer, W.M.; McCullough, M.; Karanja, N.; Lin, P.H.; Steele, P.; Proschan, M.A., et al. Rationale and design of the Dietary Approaches to Stop Hypertension trial (DASH). A multicenter controlled-feeding study of dietary patterns to lower blood pressure. *Annals of epidemiology* **1995**, *5*, 108-118, doi:10.1016/1047-2797(94)00055-x.
5. Shivappa, N.; Steck, S.E.; Hurley, T.G.; Hussey, J.R.; Hébert, J.R. Designing and developing a literature-derived, population-based dietary inflammatory index. *Public Health Nutr* **2014**, *17*, 1689-1696, doi:10.1017/S1368980013002115.
6. WHO. Healthy diet: key facts. World Health Organization: Geneva, Switzerland, 2020.
7. Willett, W.; Rockström, J.; Loken, B.; Springmann, M.; Lang, T.; Vermeulen, S.; Garnett, T.; Tilman, D.; DeClerck, F.; Wood, A., et al. Food in the Anthropocene: the EAT-Lancet Commission on healthy diets from sustainable food systems. *Lancet* **2019**, *393*, 447-492, doi:10.1016/S0140-6736(18)31788-4.
